# Characterization of the Factors that Influence Sinapine Concentration in Rapeseed Meal during Fermentation

**DOI:** 10.1371/journal.pone.0116470

**Published:** 2015-01-21

**Authors:** Yanxing Niu, Mulan Jiang, Mian Guo, Chuyun Wan, Shuangxi Hu, Hu Jin, Fenghong Huang

**Affiliations:** 1 Oil Crops Research Institute, Chinese Academy of Agricultural Sciences, Wuhan, Hubei Province, P.R.China; 2 Hubei Key Laboratory of Lipid Chemistry and Nutrition, Wuhan, Hubei Province, P.R.China; University of Nottingham, UNITED KINGDOM

## Abstract

We analyzed and compared the difference in sinapine concentration in rapeseed meal between the filamentous fungus, *Trametes* sp 48424, and the yeast, *Saccharomyces cerevisiae*, in both liquid and solid-state fermentation. During liquid and solid-state fermentation by *Trametes* sp 48424, the sinapine concentration decreased significantly. In contrast, the liquid and solid-state fermentation process by *Saccharomyces cerevisiae* just slightly decreased the sinapine concentration (P ≤ 0.05). After the solid-state fermented samples were dried, the concentration of sinapine in rapeseed meal decreased significantly in *Saccharomyces cerevisiae*. Based on the measurement of laccase activity, we observed that laccase induced the decrease in the concentration of sinapine during fermentation with *Trametes* sp 48424. In order to eliminate the influence of microorganisms and the metabolites produced during fermentation, high moisture rapeseed meal and the original rapeseed meal were dried at 90°C and 105°C, respectively. During drying, the concentration of sinapine in high moisture rapeseed meal decreased rapidly and we obtained a high correlation coefficient between the concentration of sinapine and loss of moisture. Our results suggest that drying and enzymes, especially laccase that is produced during the solid-state fermentation process, may be the main factors that affect the concentration of sinapine in rapeseed meal.

## Introduction

Rapeseed is the second largest oilseed crop in the world and accounts for 12.6% of the oilseed production [1]. Rapeseed meal is the most important by-product of rapeseed processing and is a good source of protein [2]. Although rapeseed meal is well balanced in essential amino acids [3], it also contains antinutrients, such as glucosinolates, phenolic compounds and phytic acid, which limit its application for feed and food [4–6].

Among the various antinutrients, sinapic acid is the predominant phenolic acid in rapeseed accounting for about 73% of the free phenolic acids, and present at a frequency of 1 to 2% in rapeseed [7, 8]. However, most of sinapic acid is represented as the form of sinapine in rapeseed. As a choline ester, sinapine is a bitter tasting compound and contributes to the unpleasant taste of rapeseed meal and protein products [8]. Furthermore, feeding laying hens with rapeseed meal that contains sinapine may result in the production of eggs with a fishy odor [9].

In order to remove antinutrients, many methods such as solvent extraction, chemical degradation, enzyme hydrolysis, and fermentation have been utilized [10–12]. Fermentation is regarded as a good method of detoxification and improving the protein concentration [13]. For example, fermentation with *Rhizopus oligosporus* sp T3 for 10 h, resulted in the degradation of 84% of carbohydrates, 30% of lignin, and 47% of total glucosinolates in rapeseed meal [14].

It has been reported that polyphenol oxidases from the fungus *Trametes versicolor* can catalyze sinapine degradation [15]. During the growth of the fungus *Pleurotus ostreatus* in solid-state fermentation, laccase was produced and sinapine was degraded [5]. Currently, bacteria, yeast are also used for the fermentation of rapeseed meal [13]. Except for Basidiomycota fungus, their influence on sinapine and the factors that cause the changes in the concentration of sinapine are still unknown. It has been reported that several microorganisms may decrease the concentration of sinapine in rapeseed meal [16]. In our previous research, we found that certain microorganisms have a great influence on the concentration of sinapine during the solid-state fermentation process, while they have almost no effect on the sinapine concentration during liquid fermentation.

In order to further understand the factors that affect the concentration of sinapine in rapeseed meal during fermentation, yeast and Basidiomycota fungus were cultured in media that contained sinapine. The goals of the current study are as follows:

1. To evaluate the effect of the chosen yeast and Basidiomycota fungus samples on the concentration of sinapine during fermentation,
2. To analyze the factors that may affect the concentration of sinapine in rapeseed meal during fermentation.

## Materials and Methods

### Chemicals

Methanol, peptone, yeast extract, glucose, and malt extract were purchased from Sinopharm Chemical Reagent Co. Ltd., Shanghai, China. Standard sinapine thiocyanate was obtained from the National Institute for the Control of Pharmaceutical and Biological Products, China. Laccase was purchased from Sigma (Sigma-Aldrich Co. LLC. MO, USA).

### Apparatus

The UV-VIS Spectrometer, Specord 200, was obtained from Analytik Jena AG, Jena, Germany. The grinder, 1095 Knifetec Sample Mill was procured from Foss Tecator, Höganäs, Sweden. The ultrasonic cleaner, SB120DT, was obtained from Ningbo Scientz Biotechnology Co., Ltd., Zhejiang, China. The shaker, HYL-C multi-function plain bumper was procured from Taicang Qiang Le Laboratory Equipment Co., Ltd. Shanghai Jing Hong Laboratory Instrument Co., Ltd., Shanghai, China provided the DHG-91468 heating and drying oven. The ultra-performance liquid chromatograph, Acquity UPLC, was equipped with a degasser, two solvent delivery modules, an autosampler, a column oven, and a DAD (Waters Corporation, MA, USA).

### Samples

Rapeseed meals were obtained from the Hubei Allstar Oil and Grains Industrial Company, Hubei, China.

Sinapine thiocyanate was extracted from rapeseed meal [17] and isolated by the method modified from Qiao [18]. After ground for 20 s, the samples were weighed in a flask and 15 equivalent volumes of hot methanol (purity > 99%) were added (v•w^−1^). The samples in the capped flask were extracted twice under ultrasonication at 60°C. The extracted solutions were collected in another flask and evaporated to form a syrup by rotary evaporators. The syrup was diluted with water, excess potassium thiocyanate (KSCN, 20% in aqueous solution) was added, and the syrup was stored in a fridge for 48 h at 4°C. The precipitates were then resolved in 40 equivalent volumes of hot 95% ethyl alcohol (w•v^−1^), and recrystallized twice. The crystals were dried and sinapine thiocyanate was obtained. Its purity was 95.43% as measured by HPLC and based on the method by Niu et al [17].

### Strains

*Trametes* sp 48424, a Basidiomycota fungus and the yeast *Saccharomyces cerevisiae*, were utilized for the current study. It has been reported that polyphenol oxidase catalyzes sinapine in rapeseed. *Trametes* sp 48424 has been reported to be capable of generating high yields of laccase [19], a kind of polyphenol oxidase which catalyzes single-electron oxidations of phenolic compounds [20]. Yeast has also been used in rapeseed fermentation [13]. Thus, *Trametes* sp 48424 and *Saccharomyces cerevisiae* were chosen to evaluate the effect of fermentation, by different strains, on the concentration of sinapine in rapeseed meal.

*Trametes* sp 48424 was obtained from the School of Life Science and Technology, Huazhong University of Science and Technology. *Saccharomyces cerevisiae* was isolated from glutinous rice wine. They were maintained on agar slants at 4°C [19].

### Seed culture

The medium used for *Saccharomyces cerevisiae* seed culture had the following composition: peptone (2 g), yeast extract (1 g), glucose (2 g), malt extract (1 g), and water (100 mL). For the seed culture, *Saccharomyces cerevisiae* from a fresh slant were inoculated into 30 mL of seed medium in 250 mL flasks and cultured in a rotary shaker at 180 rpm for 24 h at 28°C.

Potato medium was used for the *Trametes* sp 48424 seed culture. A loop of *Trametes* sp 48424 cells from the fresh potato dextrose agar (PDA) plate was inoculated into a 250 mL flask containing 30 mL of the liquid potato medium at 28°C with agitation at 180 rpm. Following growth for 48 h, the *Trametes* sp 48424 in the potato liquid medium was used as a seed culture.

### Liquid fermentation

Medium containing 0.1% of sinapine thiocyanate (w•w^−1^) was used for *Saccharomyces cerevisiae* liquid fermentation. Potato medium with 0.1% extracted sinapine was used for *Trametes* sp 48424 liquid fermentation. The inoculation sizes for both the strains were 10% (v•v^−1^). Both the strains were incubated in 250 mL flasks containing 30 mL of liquid medium in a rotary shaker at 180 rpm for 120 h at 28°C. During the fermentation, 1 mL each of the inoculated samples were taken for off-line analysis.

### Solid-state fermentation conditions

The medium for solid-state fermentation was prepared in 250 mL flasks by mixing 30 g of rapeseed meal and 45 mL of distilled water. The flasks with media were autoclaved and cooled down to room temperature. After inoculation, the flasks were kept in a stationary incubator at 28°C. About 10 g samples were taken out during fermentation at a given time.

Five hundred milligrams of the samples were weighed in a 50 mL capped test tube and 25 mL of hot methanol was added. The tubes were centrifuged for 20 mins by using ultrasonication at 60°C. The centrifuged supernatant was then taken out for further analysis. Four grams of sample were weighed for moisture analysis. Following solid-state fermentation, the products were dried as per routine procedure [21]. Part of the samples were dried in the oven and analyzed.

### Drying

In order to eliminate the scrambling of different strains on drying, wet rapeseed meal and original rapeseed meal were used for drying, respectively. High moisture rapeseed meal (each gram is composed of 0.4 g of water and 0.6 g of rapeseed meal) and original rapeseed meal were spread on culture dishes and dried at 90°C and 105°C, respectively. During drying, the samples were taken out to analyze the concentration of sinapine and the moisture content.

### Analysis of Samples

Samples were analyzed to quantify the laccase activity, the concentration of sinapine, and the moisture content.

The laccase activity was determined in a system containing 2.5 mL of acetic acid—sodium acetate buffer (pH 5.0), 0.4 mL of 1 mM 2,2′-azino-bis(3-ethylbenzothiazoline-6-sulphonic acid) (ABTS), and 0.1 mL of enzyme samples. After vigorous stirring, the mixture was measured at OD 420 nm. One unit of enzyme activity was defined as the amount of enzyme that catalyzes the substrate and induces an increase of 0.001 absorbance value per min.

The concentration of sinapine was measured at 326 nm using a UV-VIS Spectrometer. The calibration curves for the standard, sinapine thiocyanate is Y = 60.12×−0.00729 with determination coefficients of R^2^ = 0.9987. The calculated values of the relative standard deviations (RSD = 1.8–2.1%, n = 5) indicate reasonable repeatability of the UV method. Moreover, the limits of quantification ranged between 0.0034–0.0134 mg•mL^−1^.

### Statistical analysis

The experiments were repeated 3 times with 3 replicates for each treatment. The results were expressed as mean values and standard deviations, and the results were compared by one way analysis of variance (ANOVA) and Tukey’s test by the honest significant difference test at P = 0.05.

Data were analyzed using the SAS 9.1 (SAS Institute Inc., NC, USA) statistical software. Figures were drawn with Origin 8.0 (OriginLab Corporation, MA, USA).

## Results and Discussion

### Effect of different strains on the concentration of sinapine during liquid fermentation

**Figure 1:**
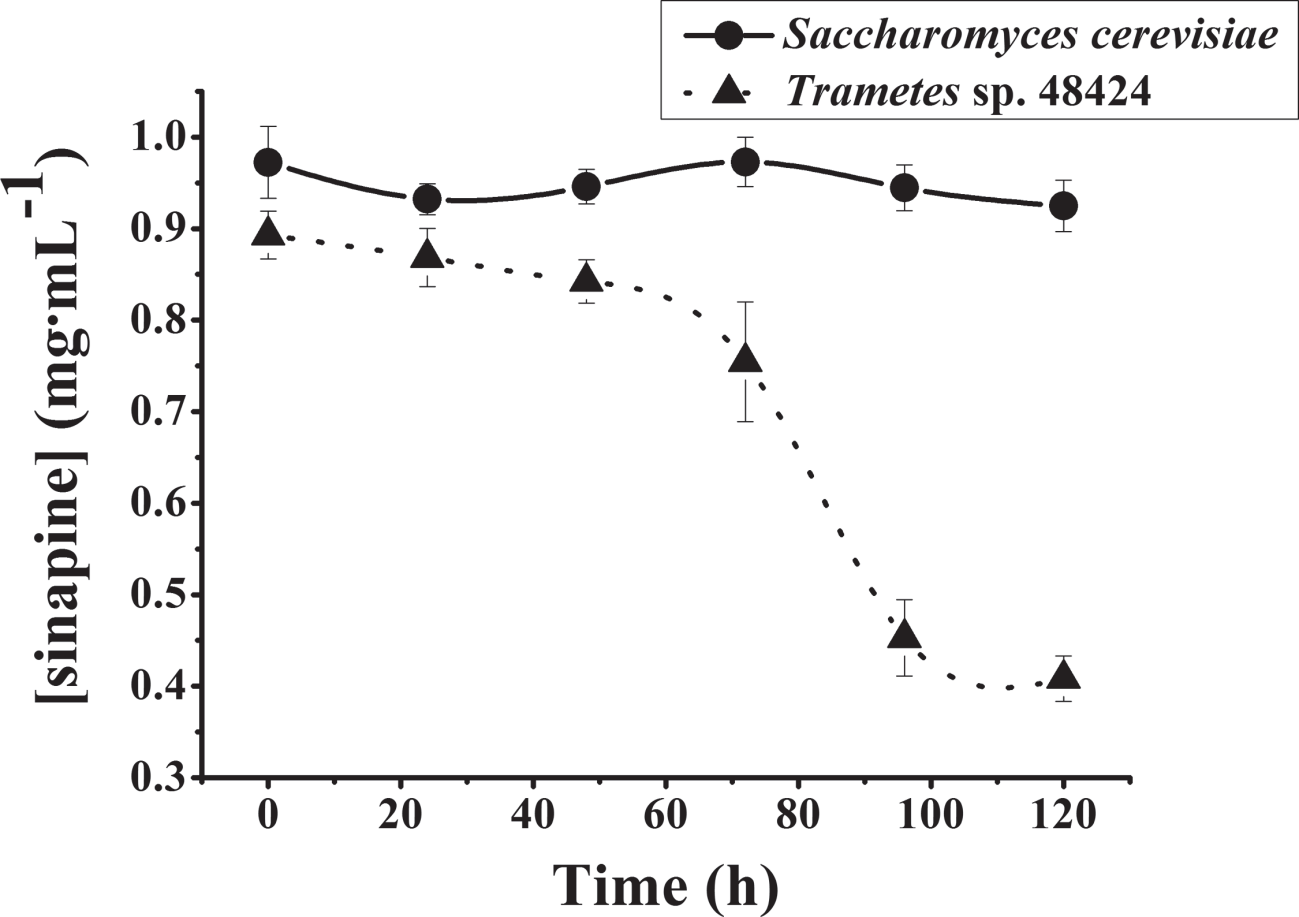
Effect of different strains on the concentration of sinapine during liquid fermentation.

Fig. 1 illustrates the changes in sinapine concentration during liquid fermentation by either *Trametes* sp.48424 or *Saccharomyces cerevisiae*. In order to maintain proper liquid circumstances, 95% purity of sinapine thiocyanate extracted from rapeseed meal was used. The initial concentration of sinapine from the two strains was similar, with the concentration being 0.97 mg•mL^−1^ and 0.89 mg•mL^−1^ for *Saccharomyces cerevisiae* and *Trametes* sp.48424, respectively. However, these values following 5 days of fermentation, reached 0.92 mg•mL^−1^ by *Saccharomyces cerevisiae* fermentation and 0.41 mg•mL^−1^ by *Trametes* sp.48424 fermentation. This result showed that the concentration of sinapine did not change significantly during liquid state fermentation by *Saccharomyces cerevisiae* (P ≤ 0.05). However, the sinapine concentration changed drastically (P ≤ 0.05) following fermentation by *Trametes* sp 48424 from 1 to 5 days, where the concentration decreased by 55%.

These results indicate that the metabolites during liquid state fermentation by *Saccharomyces cerevisiae* could not induce sinapine transformation, while the metabolites obtained during fermentation by *Trametes* sp. 48424 may induce the transformation of sinapine.

### Effect of different strains on the concentration of sinapine during solid-state fermentation

**Figure 2:**
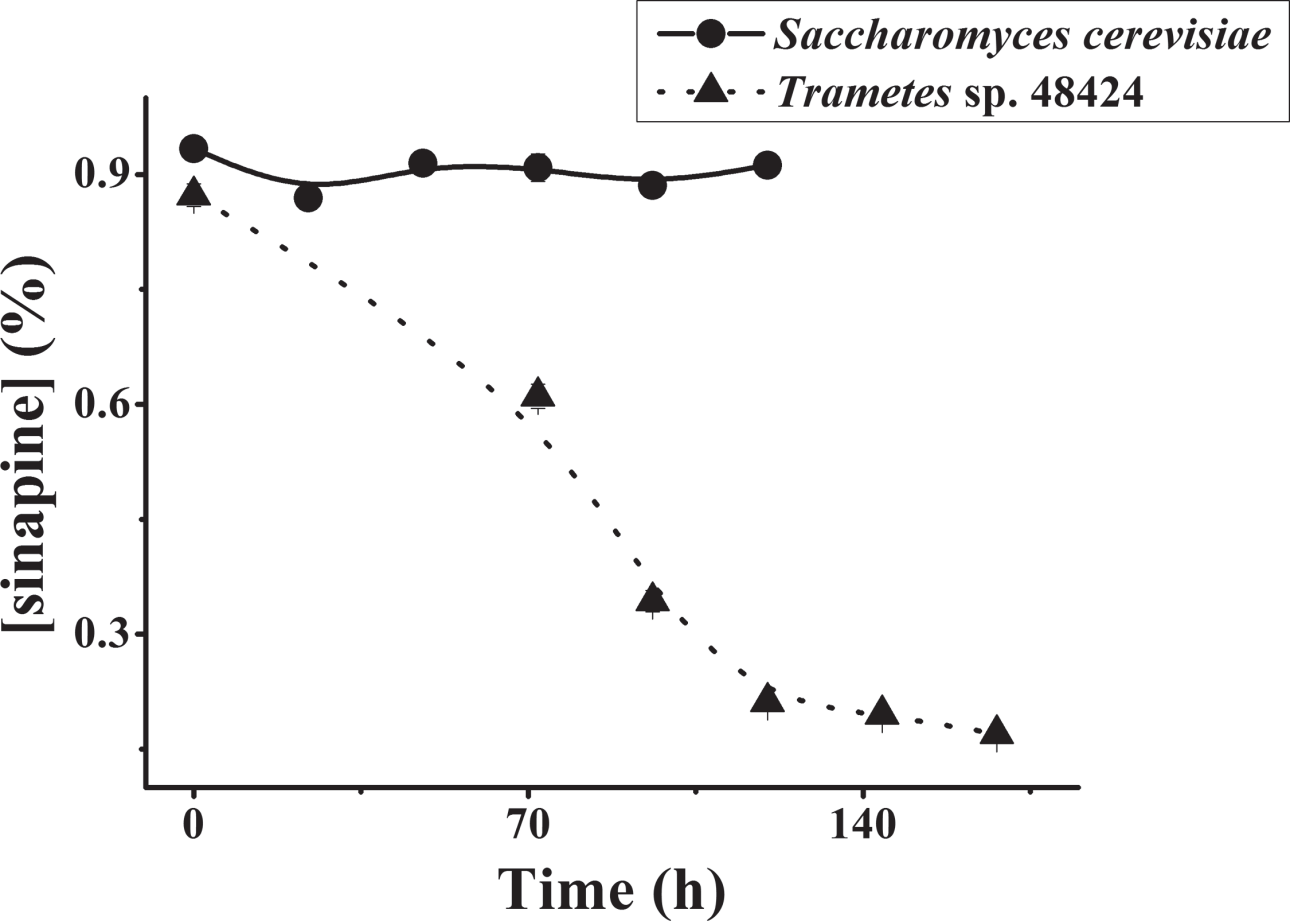
Effect of different strains on the concentration of sinapine during solid-state fermentation (samples analyzed directly and transformed as dry samples).

Fig. 2 shows the change in the concentration of sinapine during solid-state fermentation by either *Trametes* sp 48424 or *Saccharomyces cerevisiae*. In general, following solid-state fermentation, the products are dried. In our previous study, we analyzed dried product samples of solid-state fermentation. In contrast, in order to eliminate the scrambling of drying, the wet samples were analyzed directly in this study. It showed that the concentration of sinapine during solid state fermentation by *Saccharomyces cerevisiae* did not change significantly (P ≤ 0.05) from 1 to 5 days, as observed in liquid state fermentation. However, fermentation by *Trametes* sp 48424 changes the concentration of sinapine significantly (p ≤ 0.05), where the concentration decreased by 75.72% after 5 days.

The samples fermented by *Saccharomyces cerevisiae* were dried at 90°C and then analyzed. The sinapine content of dried samples was 0.24%, displayed a significant decrease compared with the samples before drying. The result showed that drying may also induce a decrease in sinapine concentration.

During the solid-state fermentation process, the products are dried by heat or lyophilization. Rozan et al [14] dried the solid fermented rapeseed meal by *Rhizopus Oligosporus* sp T3 through lyophilization, while Vig et al [21] dried *Rhizopus Oligosporus*-fermented rapeseed meal in a 60°C oven. However, the effect of drying on the concentration of sinapine has never been investigated. It is still elusive whether heat or enzymes in the rapeseed meal are factors that affect the concentration of sinapine.

### Effect of laccase on the concentration of sinapine during liquid fermentation

**Figure 3:**
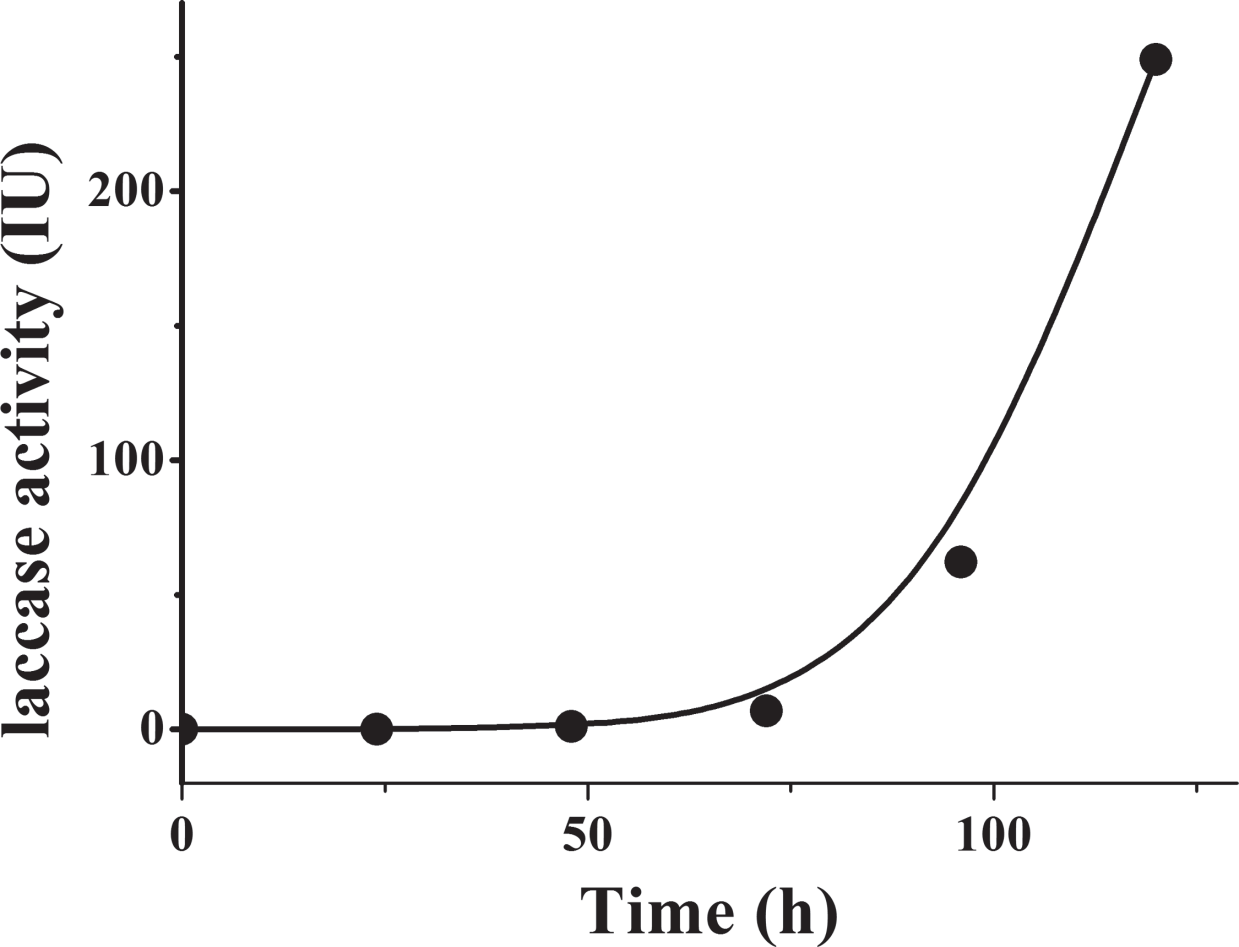
Changes in laccase activity during liquid fermentation by *Trametes* sp.48424.

The activity of laccase increased during liquid state fermentation by *Trametes* sp 48424 is shown in Fig. 3. After 2 days of fermentation, the activity of laccase in *Trametes* sp 48424 solution could be detected. Following 2 days of fermentation, the activity increased quickly and reached 249 IU. The laccase activity in *Saccharomyces cerevisiae* solution was also measured but not detectable during the liquid state fermentation.

Fifty microlitres of *Trametes* sp 48424-incubated solution that was fermented for 7 days in the absence of sinapine, and a 0.1 mg•mL^−1^ laccase solution (≥ 1000 unit•mg^−1^, Sigma-Aldrich Co. LLC. MO, USA) were added to a 2 mL of 0.1 mg•mL^−1^ sinapine solution respectively, and analyzed to determine their effect on the concentration of sinapine. Both the solutions gradually became darker. During the first 2 minutes, the color change could be seen and the concentration of sinapine decreased to 0.06 mg•mL^−1^ in the *Trametes* sp 48424 solution.

According to Fig. 1, Fig. 3, and the phenomenon above, it can be confirmed that the enzyme produced during fermentation was the key reason for the decrease of the concentration of sinapine in samples fermented by *Trametes* sp 48424. Though all the microorganisms secrete enzymes, only specific enzymes have an effect on sinapine. Laccase is an appropriate enzyme that catalyzes sinapine, resulting in its transformation. According to Qiao [19], there are several enzymes that can transform sinapine, such as tyrosinase and tannase. Tyrosinase is capable to breakdown sinapine, while tannase and ferulic acid esterase hydrolyze sinapine. Polyphenol oxidase is also an important enzyme that catalyzes sinapine according to Lacki and Dunvjak [15]. The mechanism of the initial rate of sinapine transformation by polyphenol oxidase has been described based on the Theorell-Chance Bi-Bi mechanism. Laccases (EC1.10.3.2) belong to polyphenol oxidases that are usually obtained from plants, insects, bacteria, and fungi, especially white-rot fungi [22]. They are multi-copper containing oxidoreductases that catalyze the monoelectronic oxidation of various substrates [23]. In addition, laccase is a kind of particular polyphenol oxidase that has a broad substrate specificity. It can catalyze a wide range of substrates, including phenols, polyphenols, anilines, aryl diamines, methoxy-substituted phenols, hydroxyindoles, benzenethiols, and inorganic/organic metal compounds [20]. Hu et al [5] reported that the fungus *Pleurotus ostreatus* DHOM 197961 is able to grow well in canola meal, and during solid-state fermentation it can produce laccase that decreases sinapic acid esters. The results are similar to those from liquid fermentation by *Trametes* sp 48424.

However, it is worth noting that the decrease in the concentration of sinapine (Fig. 1) that is transformed by *Trametes* sp 48424 is not highly consistent with the increase in laccase activity (Fig. 3). A significant decrease in the concentration of sinapine occurred during 70 to 90 h, as shown in Fig. 1, while a dramatic increase in laccase activity occurred after 90 h of fermentation in Fig. 3. This is ascribed to the potential high efficiency of laccase that showed very fast rate constants in related enzymatic transformations [15]. As a result, once laccase was generated in the fermentation system, the enzymatic degradation of sinapine occurred immediately (70 h in both Fig. 1 and Fig. 3). In addition, due to high efficiency, the low concentration of laccase is sufficient to promote the total transformation of sinapine. Hence, the reaction terminated prior to the dramatic accumulation of laccase.

The above results demonstrate that different strains have a distinct influence on the concentration of sinapine in rapeseed meal during fermentation, and this is associated with enzymes, such as laccase. *Trametes* sp 48424 was effective in decreasing the concentration of sinapine during liquid and solid-state fermentation because of its production of laccase, while *Saccharomyces cerevisiae* has no effect on sinapine in rapeseed meal during liquid and solid-state fermentation due to the absence of laccase production.

### Effect of drying on the concentration of sinapine in rapeseed meal

**Figure 4:**
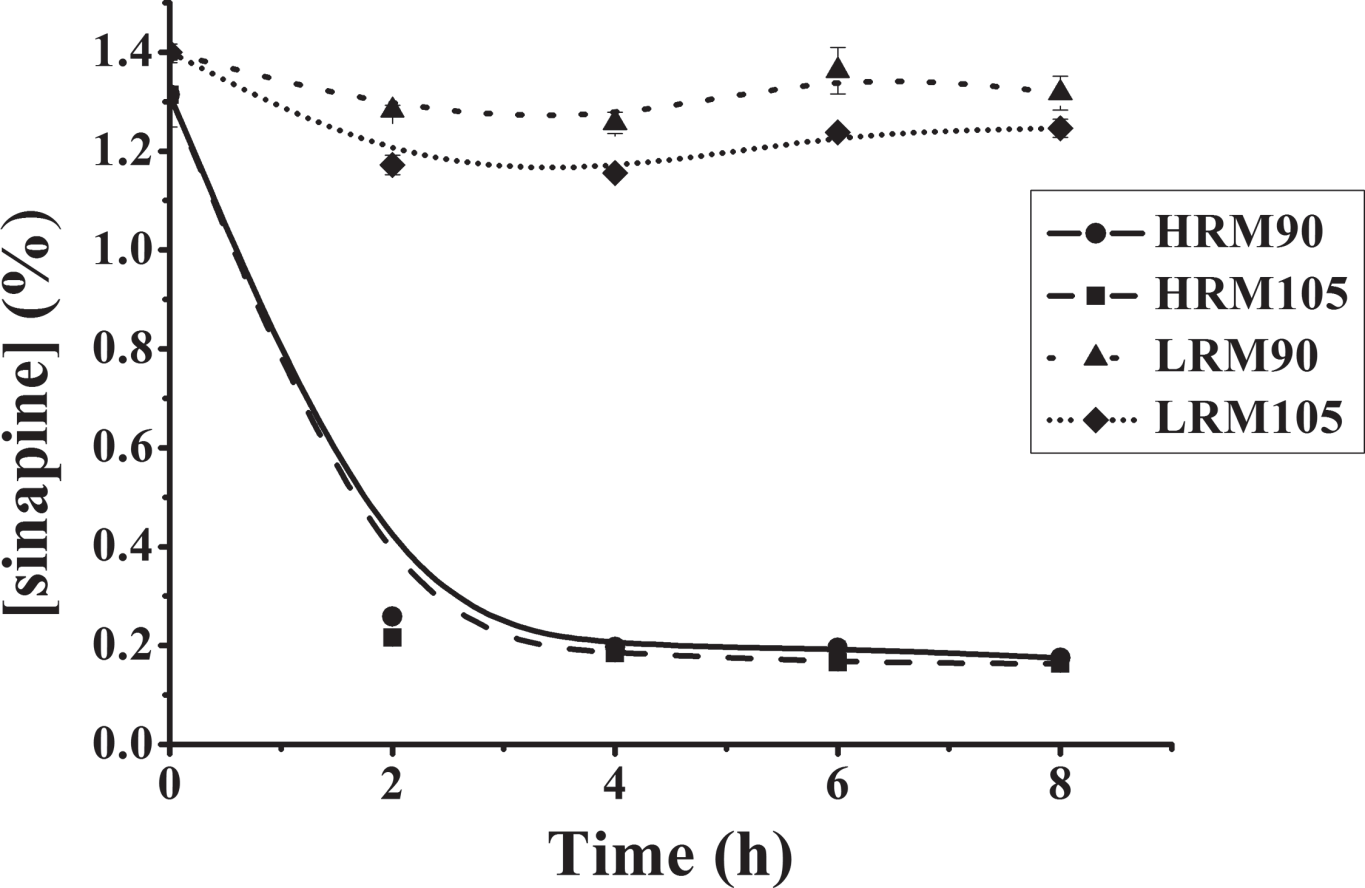
Effect of drying on the concentration of sinapine in rapeseed meal (HRM90, high moisture rapeseed meal dried at 90°C; HRM105, high moisture rapeseed meal dried at 105°C; LRM90, original rapeseed meal dried at 90°C; LRM105, original rapeseed meal dried at 105°C).

From the above results, drying can induce the decrease in the concentration of sinapine. In order to eliminate the influence of microorganisms and the metabolites produced during fermentation, high moisture rapeseed meal (each gram is composed of 0.4 g water and 0.6 g rapeseed meal) and original rapeseed meal (10% moisture content) were dried (Fig. 4). As observed, the concentration of sinapine in high moisture rapeseed meal decreased a lot (P ≤ 0.05) in the first 2 hours, while the decrease was not significant after 2 hours. Two sample paired t-tests were employed to analyze the effect of two different temperatures on the concentration of sinapine in the rapeseed meal. The results indicated that the concentration of sinapine in the samples was significantly influenced by temperature before 4 hours (p ≤ 0.05), while after 4 hours the concentration of sinapine varied slightly. The concentration of sinapine in the original rapeseed meal changed significantly (p ≤ 0.05) during drying, though this difference was not large.

**Figure 5:**
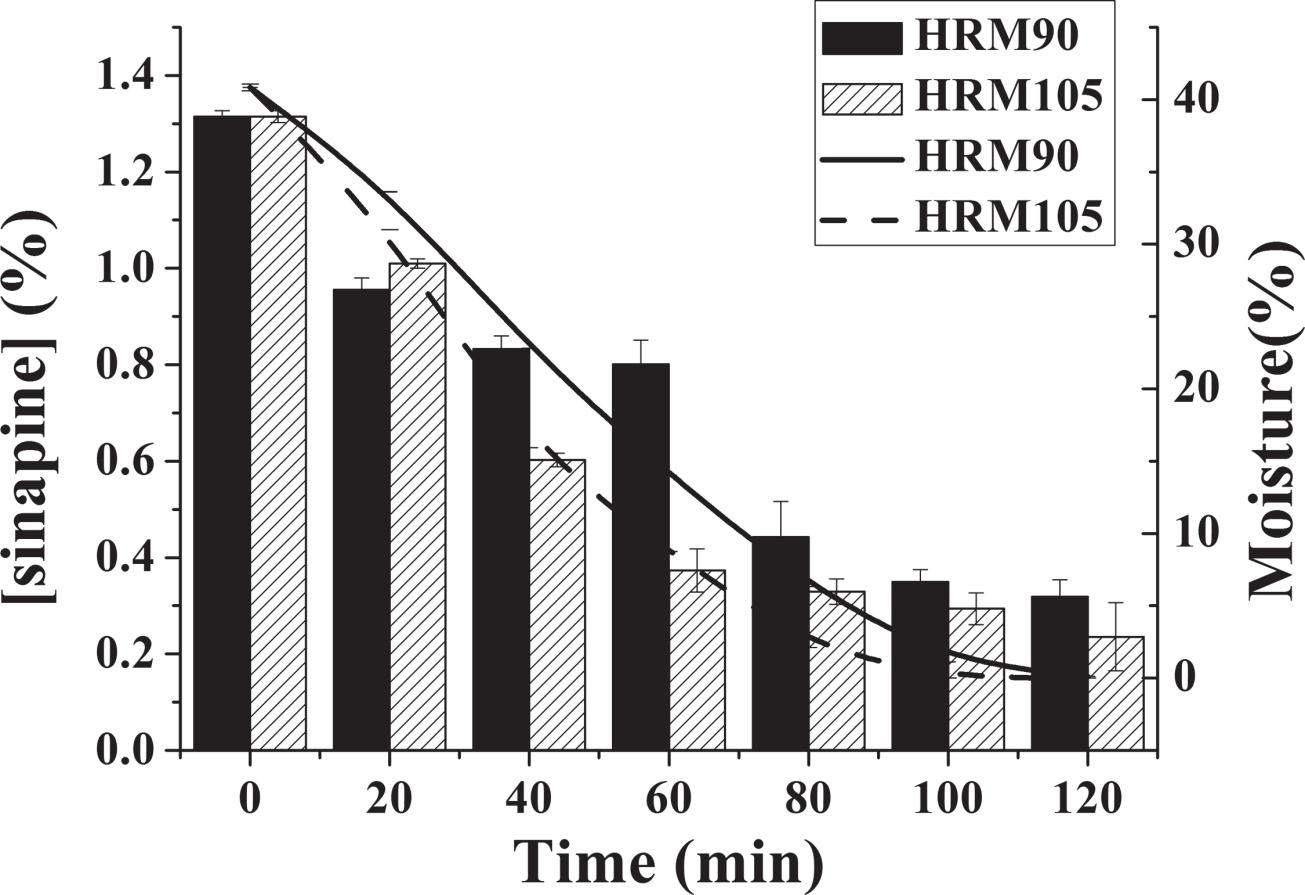
Effect of drying and moisture on the concentration of sinapine in high moisture rapeseed meals (HRM90, high moisture rapeseed meal dried at 90°C; HRM105, high moisture rapeseed meal dried at 105°C).

In order to monitor the major changes in the concentration of sinapine in the high moisture rapeseed meal during the first 2 hours, the sinapine concentration during drying was detected (Fig. 5). We observed that the concentration of sinapine decreased quickly in 2 hours accompanied by rapid loss of water. The correlation analysis was performed with SAS 9.0. The results showed that the correlation coefficient between sinapine concentration and moisture were 0.9693 and 0.9923 when dried at 90°C and 105°C, respectively. This result proved that drying may influence the concentration of sinapine in rapeseed meal.

It is reported that autoclaving has an effect on sinapic acid but has no effect on sinapine [24]. Sinapine has a decomposition point at 127°C [18], so it should be stable at 90°C and 105°C. With a low moisture content, the concentration of sinapine of rapeseed meal did not change a lot, while at a higher moisture content, the sinapine concentration decreased rapidly with a high correlation coefficient between concentration and loss of moisture. Thus, moisture appears to be a key factor that influences the concentration of sinapine during fermentation.

## Conclusion

Liquid fermentation and solid-state fermentation can both cause a decrease in the concentration of sinapine. In most situations, the decrease in sinapine concentration in rapeseed meal is induced by the laccase enzyme. Drying may be another reason for the decrease in sinapine concentration in dried products during solid fermentation. The result provides a theoretical basis for the isolation of strains and to improve the technique of rapeseed meal fermentation. In the future, the factors that influence the decrease in the concentration of sinapine in dried fermented samples, should be further discussed.
